# Pantograph Detection Algorithm with Complex Background and External Disturbances

**DOI:** 10.3390/s22218425

**Published:** 2022-11-02

**Authors:** Ping Tan, Zhisheng Cui, Wenjian Lv, Xufeng Li, Jin Ding, Chuyuan Huang, Jien Ma, Youtong Fang

**Affiliations:** 1 School of Automation and Electrical Engineering, Zhejiang University of Science and Technology, Hangzhou 310023, China; 2 College of Electrical Engineering, Zhejiang University, Hangzhou 310027, China; 3 Chinese-German Institute for Applied Engineering, Zhejiang University of Science and Technology, Hangzhou 310023, China

**Keywords:** high-speed railway, object detection, blob detection, EOR-Brenner, blur and dirt, complex background

## Abstract

As an important equipment for high-speed railway (HSR) to obtain electric power from outside, the state of the pantograph will directly affect the operation safety of HSR. In order to solve the problems that the current pantograph detection method is easily affected by the environment, cannot effectively deal with the interference of external scenes, has a low accuracy rate and can hardly meet the actual operation requirements of HSR, this study proposes a pantograph detection algorithm. The algorithm mainly includes three parts: the first is to use you only look once (YOLO) V4 to detect and locate the pantograph region in real-time; the second is the blur and dirt detection algorithm for the external interference directly affecting the high-speed camera (HSC), which leads to the pantograph not being detected; the last is the complex background detection algorithm for the external complex scene “overlapping” with the pantograph when imaging, which leads to the pantograph not being recognized effectively. The dirt and blur detection algorithm combined with blob detection and improved Brenner method can accurately evaluate the dirt or blur of HSC, and the complex background detection algorithm based on grayscale and vertical projection can greatly reduce the external scene interference during HSR operation. The algorithm proposed in this study was analyzed and studied on a large number of video samples of HSR operation, and the precision on three different test samples reached 99.92%, 99.90% and 99.98%, respectively. Experimental results show that the algorithm proposed in this study has strong environmental adaptability and can effectively overcome the effects of complex background and external interference on pantograph detection, and has high practical application value.

## 1. Introduction

**Figure 1:**
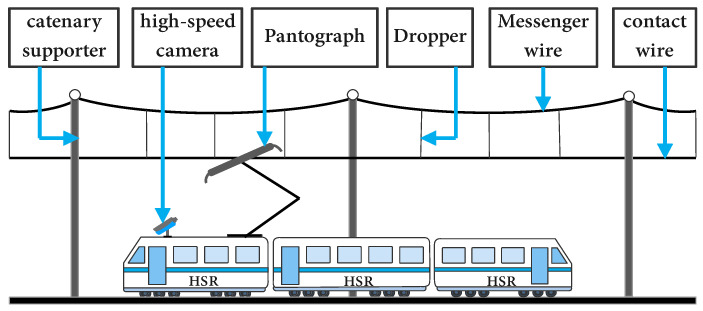
Schematic of PCS.

As an important part of the pantograph-catenary system (PCS), the pantograph is a special current-receiving device installed on the roof of the high-speed railway (HSR). When the pantograph is raised, it transmits power from the traction substation to the HSR through the friction between the pantograph and the contact network, thus providing the power required for the operation of the HSR. Once a pantograph failure occurs, it will directly affect the operational safety of HSR [1,2,3]. Therefore, the current pantograph status must be accurately assessed through real-time detection of pantographs to ensure the safety and stability of HSR operation. The PCS is shown in Figure 1.

**Figure 2:**
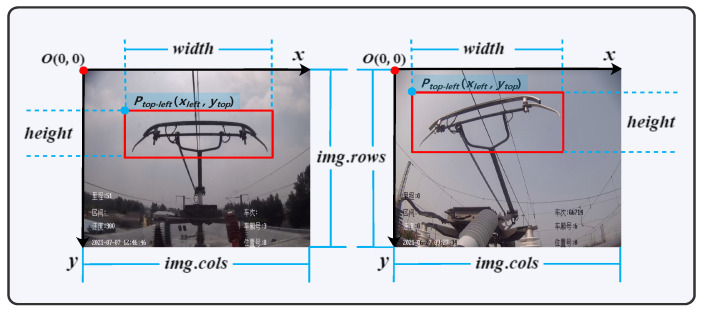
HSC footage of pantographs.

There are two main models of HSR in actual operation, the speed of the two models of HSR is usually 150–300 km/h when they are running stably, but the images captured by the high-speed cameras (HSC) equipped with the two models of HSR are slightly different. One is the image captured by HSR-A as shown in the left image in Figure 2, and the other is the image captured by HSR-B as shown in the right image in Figure 2. It is worth mentioning that there are some Chinese messages in the images captured by the HSC in Figure 2, which contain the basic information of the vehicle and the time information and do not affect the reader’s understanding of this paper. The same is true for the images captured by the relevant HSC that appear subsequently in the paper.

Although the two models of HSR are equipped with different angles of HSC, they both have a frame rate of 25 FPS. Therefore, regardless of the operating speed of HSR, the HSC can only capture 25 pantograph images per second, so the algorithm must process at least 25 images captured by the HSC per second to meet the real-time requirement. The region corresponding to the red rectangle in Figure 2 is the region of interest (ROI), and the pantograph in the ROI is the main research object of this study.

In the current pantograph detection method, Refs. [4,5] proposed the use of Catenary and Pantograph Video Monitor (CPVM-5C) System for pantograph detection, but in the 5C system the camera is generally installed at the HSR exit, which cannot detect and monitor the running HSR in real time. Refs. [6,7,8] proposed to extract the edges of pantographs by improved edge detection, wavelet transform, hough transform, etc., so as to realize the evaluation of pantographs, but this is essentially based on the traditional image processing method, which is only applicable to pantograph detection when the overall image is clear and the background is single, which is limited and difficult to meet the complex situation when the HSR is actually running. Refs. [9,10,11] proposed to achieve real-time pantograph detection by simply using a certain improved neural network, whose detection results are entirely given by the neural network. This method relies heavily on a large number of data sets for support, and is prone to a large number of false alarms when the training set is not rich enough in samples. The data set of certain complex scenes in the operation of HSR is difficult to obtain, so it is difficult to build a model that covers a large number of rich scene samples under training, which makes a large interference to the detection results when disturbed. Refs. [12,13,14,15] and others combine deep learning and image processing to greatly improve the stability of pantograph detection by a single reliance on neural networks, but there are still major limitations in complex scenes. The proposed methods of [16,17,18] are not very practical for complex scenes and external interference, and the complex scenes that can be overcome are very limited.

In the actual operation of HSR, it is often faced with various complex environments and changing scenarios. Even for HSR running on the same line, there may be huge differences in the scenarios encountered in different time periods. This difference is caused by multiple factors, which is irregular and difficult to predict. Because the occurrence of these scenes is full of randomness, resulting in a sample set for training neural networks that cannot cover all situations in all complex scenes and environments. With limited samples, methods to improve detection accuracy by improving certain neural networks do not fundamentally address the large number of pantograph state false positives in such scenarios, and cannot really address the impact of complex scenarios in the actual operation of HSR. Therefore, this paper focuses on filtering and detecting these complex scenes and external interference by designing algorithms, so as to achieve a method more in line with the actual operation of HSR and more widely applicable, reducing or even eliminating these scenes for neural network real-time detection of a pantograph’s impact.

## 2. YOLO V4 Locates the Pantograph Region

The Alexey-proposed You Only Look Once (YOLO) V4 is a huge upgrade to the one-stage detector in the field of object detection [19]. Compared with the previous version of YOLO, YOLO V4 replaces the backbone network from the original darknet53 to CPSdarknet53 on the basis of YOLO V3, which makes YOLO V4 effectively reduce the amount of computation and improve the learning ability. Meanwhile, YOLO V4 replaces spatial pyramid pooling (SPP) with feature pyramid networks (FPN), which splices feature maps at different scales and increases the receptive field of the model, enabling YOLO V4 to extract more details.

**Figure 3:**
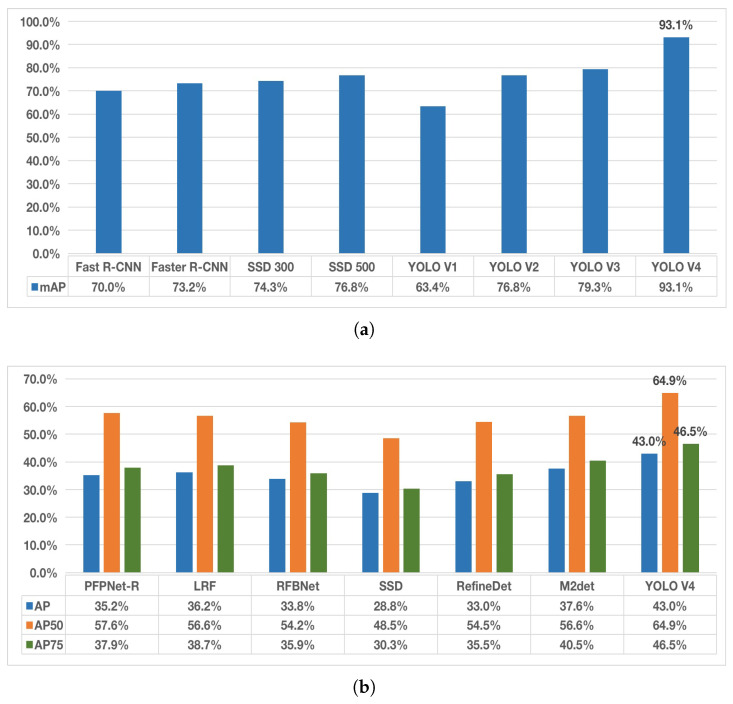
Comparison of YOLO V4 with other mainstream neural networks [20,21,22,23,24,25,26,27,28,29,30,31,32]. (**a**) Test results on VOC2007 + VOC2012. (**b**) Test results on the COCO dataset.

Average Precision (AP) and Mean Average Precision (mAP) are important metrics to measure the performance of the target detection algorithm, while AP-50 and AP-75 are the AP values when the corresponding Intersection over Union (IoU) thresholds are set to 0.5 and 0.75. The performance of YOLO V4 and current mainstream object detection algorithms on two datasets, Visual Object Classes (VOC) and Common Objects in Context (COCO), is shown in Figure 3.

**Figure 4:**
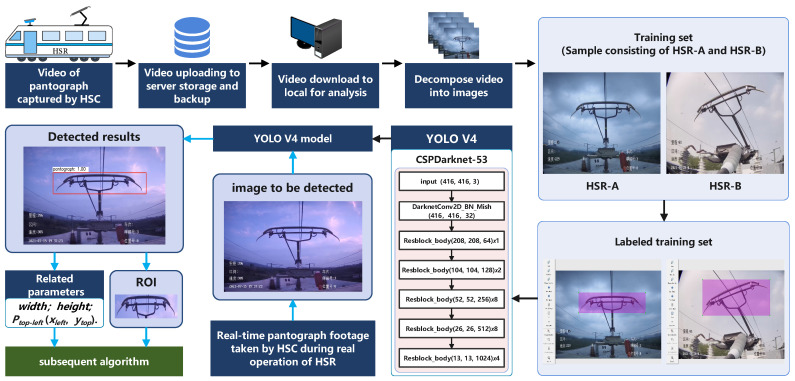
YOLO V4 overall algorithm process.

Figure 3 shows that the YOLO V4 has clear advantages in all aspects. Alexey had pointed out that the YOLO V4 was the most advanced detector at that time, and even now it still seems that the YOLO V4 has great advantages and performance [19]. Therefore, YOLO V4 is used to locate the pantograph region in this study, and the located pantograph region is passed into the subsequent algorithm. The overall algorithm flow for locating the pantograph region using YOLO V4 is shown in Figure 4.

## 3. HSC Blur and Dirt Detection Algorithm

### 3.1. Blurry HSC Screen and Dirty HSC Screen

During the operation of HSR, the HSC is always exposed to the outside of the car, which makes the HSC extremely vulnerable to interference from the outside. The external interference affecting the HSC is mainly divided into two kinds: one is the influence of rain on the pantograph during rainy days, and the other is the influence of the dirt attached to the HSC lens on the pantograph.

#### 3.1.1. Rainwater

**Figure 5:**
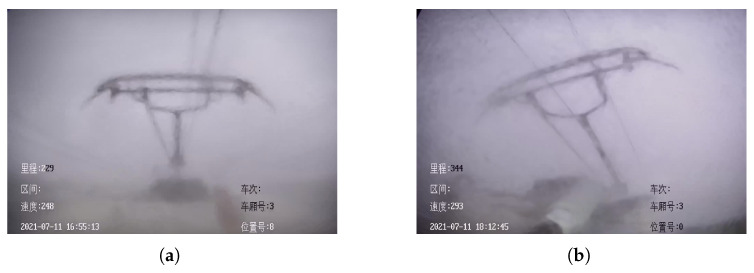
Blurred HSC imaging caused by rainwater. (**a**) HSR-A. (**b**) HSR-B.

HSR operation needs to face very complicated weather conditions, especially in rainy days, rainwater will directly affect the imaging of HSC. Figure 5 illustrates the different degrees of impact of rain on the HSR-A and HSR-B. when HSR is running at high speed, rainwater tends to cause blurring of the HSC imaging, making the captured pantographs unclear and thus causing the YOLO V4 to incorrectly assess the pantographs.

#### 3.1.2. Dirty

**Figure 6:**
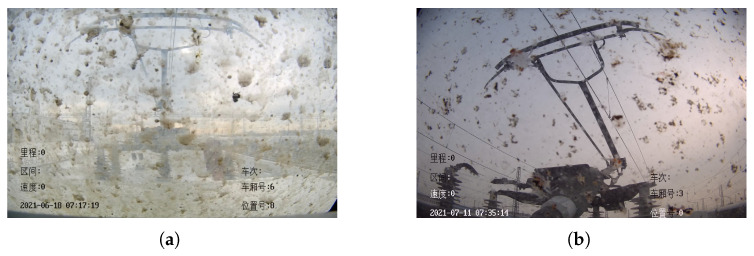
The HSC lens has a lot of dirt attached to it. (**a**) HSR-A. (**b**) HSR-B.

The lens dirt attached to the HSC can generally only be removed by manual cleaning. As shown in Figure 6, during the period from the time when the lens is dirty to before the dirt is artificially cleaned, the dirty lens will continue to affect the overall evaluation of the pantograph by YOLO V4.

### 3.2. External Factors Cause YOLO V4 to Fail to Locate the Pantograph

**Figure 7:**
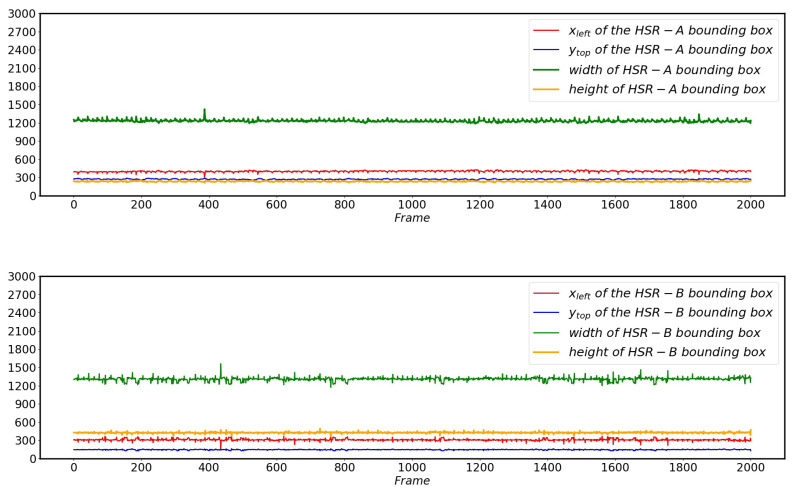
Changes of the four parameters of the bounding box when YOLO V4 is positioned normally without external interference.

When YOLO V4 cannot locate the pantograph due to external interference, the approximate position of the pantograph in the current screen can be inferred from the pantograph position that was determined in the previous normal screen. When YOLO V4 locates the pantograph area, it only needs to obtain four parameters of the bounding box in Figure 2 to achieve its accurate positioning. These four parameters are the horizontal coordinates ($x_{left}$) and vertical coordinates ($y_{top}$) of the point ($P_{top-left}$) in the upper left corner of the bounding box, and the width and height of the pantograph. The variation of the four parameters of the bounding box positioned by YOLO during normal operation of HSR of two different models is shown in Figure 7.

As can be seen from Figure 7, whether it is HSR-A or HSR-B, when its normal operation is not disturbed by external scenes, the pantograph region positioned by YOLO V4 is always relatively fixed, although there is a small range of jitter. This small-scale jitter is caused by a combination of factors such as the bumps during the operation of the HSR and the force changes between the pantograph and the catenary. This jitter does not affect the approximate position of the pantograph in the image, so when the YOLO V4 is unable to locate the pantograph area due to external interference, the approximate position of the pantograph in the current frame can be inferred from the coordinate information obtained from the previous frame, and subsequent analysis can be performed.

### 3.3. Improved Image Sharpness Evaluation Algorithm

Brenner algorithm is a classical blur detection algorithm [33], which finally achieves the evaluation of image sharpness by accumulating the square of the grayscale difference between two pixel points. Since the gray value of the image at the focal position changes significantly compared with the telefocused image, and the image at the focal position has more edge information, a more accurate judgment of the sharpness of the image can be made using this method. However, the traditional Brenner algorithm cannot meet the complex scene changes and variable external disturbances that need to be faced during the operation of high speed rail, so this paper proposes the emphasize object region-Brenner (EOR-Brenner) algorithm combined with the pantograph region localized by YOLO V4. The principle of EOR-Brenner is shown in Equation (1).

(1) $$\begin{array}{cc} F & =k_{1}F_{IMG}+k_{2}F_{ROI}\\ \; & =k_{1}\sum_{x=0}^{img.cols-3}\sum_{y=0}^{img.rows-1}{\left[f\left(x+2,y\right)-f\left(x,y\right)\right]}^{2}\\ \; & +k_{2}\sum_{x=x_{left}}^{x_{left}+width}\sum_{y=y_{top}}^{y_{top}+height}{\left[f\left(x+2,y\right)-f\left(x,y\right)\right]}^{2} \end{array}$$

where *x* is the horizontal coordinate of a pixel point, *y* is the vertical coordinate of a pixel point, f(x,y) is the gray value of the pixel point, $F_{IMG}$ and $F_{ROI}$ are the sharpness results of the corresponding region. $k_{1}$ and $k_{2}$ are the weights of the corresponding region, and *F* is the final result of the improved Brenner algorithm.

Although the ROI occupies a relatively small area of the whole image, the pantograph, as the key research object, should be given a higher weight to the area where it is located. In this study, we recommend that $k_{1}$ can be 2 or 4 times of $k_{2}$, and the specific choice should be made flexibly according to the actual operation line of HSR. After the values of $k_{1}$ and $k_{2}$ are determined, the appropriate threshold (λ) is selected based on the calculated EOR-Brenner to achieve the differentiation and detection of clear and blurred images.

As shown in (2), when the final result *F* of EOR-Brenner is higher than the set threshold (λ), the image captured by the current HSC is considered to be clear. If the pantograph cannot be detected or is detected as abnormal at this time, it can be assumed that the current detection result is not affected by the blurring of the HSC screen. However, there are still two situations: (1) the current pantograph is in normal state, although it is not affected by the blurred screen, but it may be disturbed by other external environment such as complex background, which leads to the normal pantograph being undetectable or the pantograph is incorrectly detected as abnormal. (2) The pantograph is really abnormal. At this time, it is necessary to further evaluate the real state of the pantograph through the subsequent algorithm, and finally realize the accurate detection of the real state of the pantograph.

(2) $$\begin{array}{c} \left\{\begin{array}{cc} Clear\;image, & F>\lambda \\ Blurred\;image, & F<\lambda \end{array}\right.\; \end{array}$$

### 3.4. Blob Detection Algorithm Detects Screen Dirt

When dirt is attached to a HSC, it is very easy to form blobs. Blobs caused by dirt have different areas, convexity, circularity and inertia rates, so these attributes can be used to detect and filter the blobs [34,35,36,37], and the number of blobs can ultimately determine whether the HSC is dirty or not.

The area of the blob (*S*) reflects the size of the detected blob, while the circularity derived from the area of the blob (*S*) and the corresponding perimeter (*C*) reflects the degree to which the detected spot is close to a circle, and the calculation of the circularity is shown in Equation (3):

(3) $$\begin{array}{c} Value_{circularity}=\frac{4\pi S}{C^{2}} \end{array}$$

The convexity reflects the degree of concavity of the blob. The convexity of the blob can be obtained from the area of the blob (*S*) and the area of the convex hull (*H*) of the blob, which is calculated as shown in Equation (4):

(4) $$\begin{array}{c} Value_{convexity}=\frac{S}{H} \end{array}$$

The inertia rate also reflects the shape of the blob. If an image is represented by f(x,y), then the moments of the image can be expressed by the Equation (5)

(5) $$\begin{array}{c} M_{ij}=\sum_{x}\sum_{y}x^{i}y^{j}f\left(x,y\right) \end{array}$$

For a binary image, the zero-order moment $M_{00}$ is equal to its area, so its center of mass is as shown in Equation (6):

(6) $$\begin{array}{c} \left\{\bar{x},\bar{y}\right\}=\left\{\frac{M_{10}}{M_{00}},\frac{M_{01}}{M_{00}}\right\} \end{array}$$

The central moment of the image is defined as shown in Equation (7):

(7) $$\begin{array}{c} \mu_{pq}=\sum_{x}\sum_{y}{\left(x-\bar{x}\right)}^{p}{\left(y-\bar{y}\right)}^{q}f\left(x,y\right) \end{array}$$

If only second-order central moments are considered, the image is exactly equivalent to an ellipse with a defined size, orientation and eccentricity, centered at the image center of mass and with constant radiality. The covariance moments of the image are shown in Equation (8):

(8) $$\begin{array}{c} \mathrm{cov}\left[f\left(x,y\right)\right]=\left[\begin{array}{cc} \mu_{20}^{\prime } & \mu_{11}^{\prime }\\ \mu_{11}^{\prime } & \mu_{02}^{\prime } \end{array}\right]=\left[\begin{array}{cc} \frac{\mu_{20}}{\mu_{00}} & \frac{\mu_{11}}{\mu_{00}}\\ \frac{\mu_{11}}{\mu_{00}} & \frac{\mu_{02}}{\mu_{00}} \end{array}\right] \end{array}$$

The two eigenvalues $\lambda_{1}$ and $\lambda_{2}$ of this matrix correspond to the long and short axes of the image intensity (i.e., the ellipse). Then $\lambda_{1}$ and $\lambda_{2}$ can be expressed by the Equation (9):

(9) $$\begin{array}{c} \lambda_{1}=\frac{\mu_{20}^{\prime }+\mu_{02}^{\prime }}{2}+\frac{\sqrt{4{\mu^{\prime }}_{11}^{2}+{\left(\mu_{20}^{\prime }-\mu_{02}^{\prime }\right)}^{2}}}{2}\\ \lambda_{2}=\frac{\mu_{20}^{\prime }+\mu_{02}^{\prime }}{2}-\frac{\sqrt{4{\mu^{\prime }}_{11}^{2}+{\left(\mu_{20}^{\prime }-\mu_{02}^{\prime }\right)}^{2}}}{2} \end{array}$$

The final inertia rate is obtained as shown in Equation (10):

(10) $$\begin{array}{c} Value_{inertia}=\frac{\lambda_{2}}{\lambda_{1}}=\frac{\mu_{20}^{\prime }+\mu_{02}^{\prime }-\sqrt{4{\mu^{\prime }}_{11}^{2}+{\left(\mu_{20}^{\prime }-\mu_{02}^{\prime }\right)}^{2}}}{\mu_{20}^{\prime }+\mu_{02}^{\prime }+\sqrt{4{\mu^{\prime }}_{11}^{2}+{\left(\mu_{20}^{\prime }-\mu_{02}^{\prime }\right)}^{2}}}\\ =\frac{\mu_{20}+\mu_{02}-\sqrt{4\mu_{11}^{2}+{\left(\mu_{20}-\mu_{02}\right)}^{2}}}{\mu_{20}+\mu_{02}+\sqrt{4\mu_{11}^{2}+{\left(\mu_{20}-\mu_{02}\right)}^{2}}} \end{array}$$

**Figure 8:**
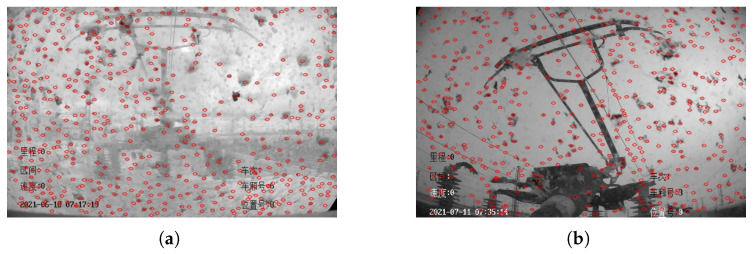
The HSC Screen dirty detection results. (**a**) HSR-A. (**b**) HSR-B.

The final selection of the number of blobs is achieved by the area, convexity, circularity and inertia rate of the blobs, and when the final number of detected blobs is greater than the set threshold, it can be inferred that the HSC surface is attached to the dirty at this time, so as to achieve the detection of HSC dirty. For the case shown in Figure 6 the final detection result is shown in Figure 8.

### 3.5. Overall Process of HSC Blur and Dirt Detection Algorithm

**Figure 9:**
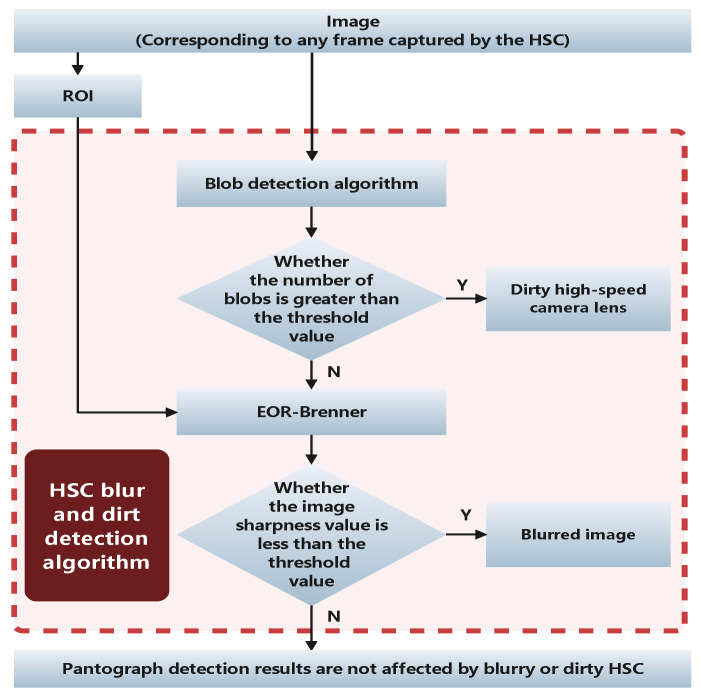
HSC blur and dirt detection algorithm process flow chart.

As shown in Figure 9, the number of blobs in the current frame is first detected by the blob detection algorithm, and when the number is greater than the set threshold it is determined that the reason why YOLO V4 cannot achieve positioning in the current frame is due to dirt, and if the number of detected spots is less than the threshold value, the EOR-Brenner is used to evaluate whether the current frame is blurred or not. Finally correctly evaluate whether the pantograph detection abnormality in the current frame or the pantograph cannot be detected is caused by the dirty and blurred HSC.

## 4. HSR Complex Background Detection Algorithm

### 4.1. The Complex Background That HSR Needs to Face

HSR often needs to face a large number of external scene changes and variable terrain, environment and other influences during actual operation. These external scenes and terrain, environment, etc. can directly affect the algorithm’s correct assessment of the real state of the pantograph, and thus a large number of false alarms occur. Compared with blur and dirt, which directly affect the HSC and thus affect the detection of pantographs, when these external scenes and terrain environments affect the detection of pantographs, the images captured by the HSC are still very clear and free of blobs, but their impact on pantograph detection is mainly due to the HSC imaging when these external disturbances and pantograph “overlap” together, thus causing a large number of false alarms on the pantograph state. In this study, we refer to this type of interference as the “complex background”, and the common complex backgrounds are catenary support devices, the sun, bridges, tunnels, and platforms of HSR.

In this study, we propose a HSR complex background detection algorithm to achieve accurate detection of these complex scenes during the operation of HSR, so as to exclude the influence of these complex background on the pantograph state evaluation.

#### 4.1.1. Catenary Support Devices

**Figure 10:**
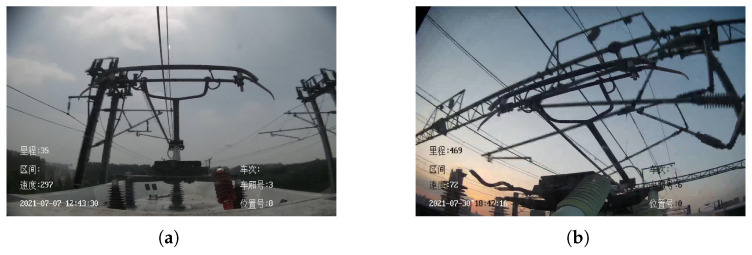
Catenary support device affects pantograph detection. (**a**) HSR-A. (**b**) HSR-B.

As an extremely important part of the whole huge HSR system, the catenary support device not only plays the role of electrical insulation, but also bears a certain mechanical load. The contact network support device, as the most frequently appearing background, as shown in Figure 10 will often affect the normal detection of pantographs.

#### 4.1.2. Sun

**Figure 11:**
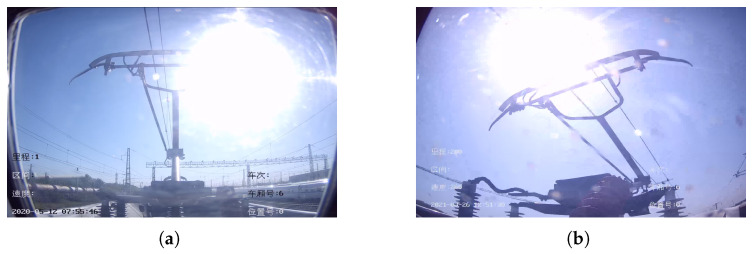
Sun affects pantograph detection. (**a**) HSR-A. (**b**) HSR-B.

As shown in Figure 11, when the sun appears in the pantograph imaging region, the strong light causes a “partial absence”-like phenomenon in the pantograph.

#### 4.1.3. Bridge

**Figure 12:**
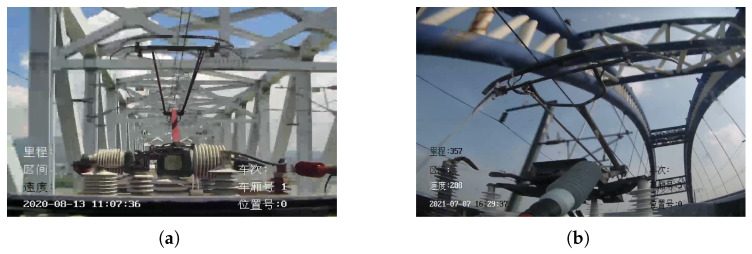
Bride affects pantograph detection. (**a**) HSR-A. (**b**) HSR-B.

Due to the complex geographical environment, when two areas are separated by rivers, only special or mixed-use bridges can be built over the rivers to provide HSR access. In more and more cities, numerous viaducts are being built to provide access to HSR. When the HSR crosses the bridge, it directly affects the detection and positioning of the pantographs. The effect of bridges on pantographs is shown in the Figure 12.

#### 4.1.4. Tunnel

**Figure 13:**
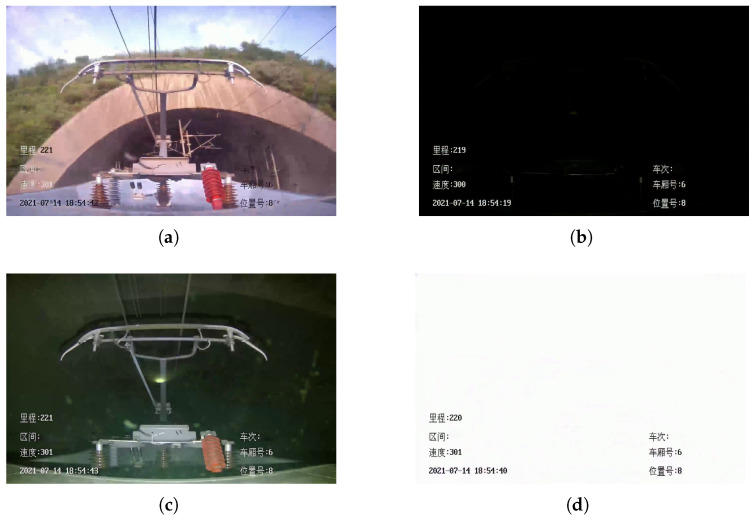
Tunnels affects pantograph detection. (**a**) Before the HSR enters the tunnel. (**b**) The moment the HSR enters the tunnel. (**c**) After the fill light is turned on, the HSR runs stably in the tunnel. (**d**) The moment the HSR exits the tunnel.

The presence of the tunnel greatly reduces the travel time and shortens the mileage between the two areas. Figure 13 shows the different images captured by the HSC before and after the HSR enters the tunnel. When the HSR enters the tunnel and runs stably, as shown in Figure 13c, the normal monitoring of the pantograph can still be achieved at this time because the fill light on the HSR is turned on. However, as shown in Figure 13b and Figure 13d, the dramatic light changes during the short period of time when the HSR enters and leaves the tunnel will cause the neural network to fail to achieve accurate positioning and detection of the pantographs when entering and leaving the tunnel.

#### 4.1.5. Platform

**Figure 14:**
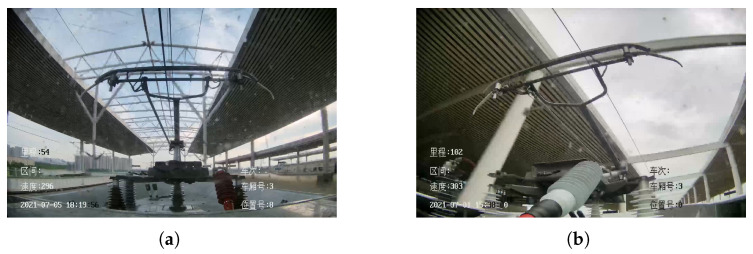
Platform affects pantograph detection. (**a**) HSR-A. (**b**) HSR-B.

As shown in Figure 14, when the HSR drives into the platform, the platform will partially overlap with the pantograph region, which affects YOLO’s positioning and detection of the pantograph, thus causing a large number of false alarms of the pantograph status by YOLO in the platform.

### 4.2. Tunnel Detection Algorithm Based on the Overall Average Grayscale of the Image

For such false alarms caused by drastic changes in light over a short period of time that cause YOLO to be unable to detect and locate the pantograph for a short period of time, they can be excluded by the grayscale change rule of the image. The average grayscale calculation method of the image is shown in Equation (11):

(11) $$\begin{array}{c} \bar{g}=\frac{\sum_{i=0}^{img.cols-1}\sum_{j=0}^{img.rows-1}P\left(i,j\right)}{img.cols*img.rows} \end{array}$$

where P(x,y) is the grayscale of the corresponding pixel point, img.rows is the height of the image and img.cols is the width of the image.

**Figure 15:**
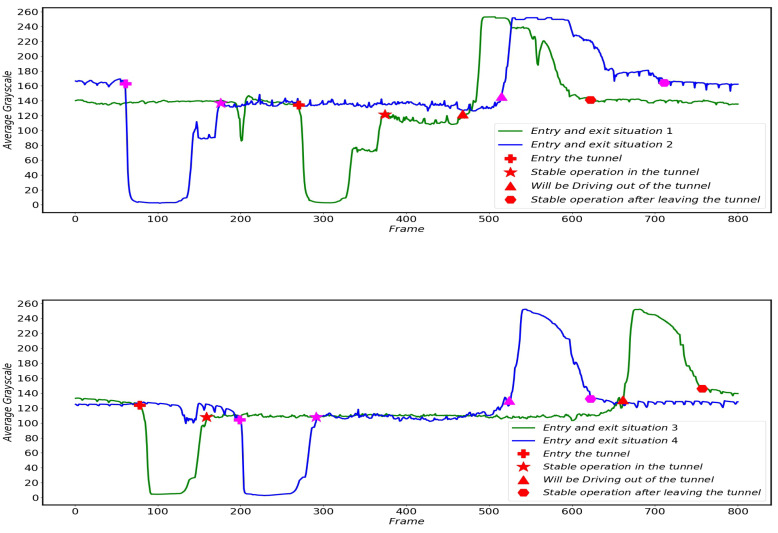
Average grayscale variation of images of HSR-A (**top**) and HSR-B (**bottom**) when driving into different tunnels.

When the pantograph is running in a relatively clear and clean background, the image corresponding to each frame will cause the average grayscale of the image to fluctuate in a small range with the continuous operation of the HSR and the continuous change of the scene, but there will not be a large change in the average grayscale. Figure 15 shows the change in the average grayscale of the images taken by the HSC before and after the different cars enter and exit the tunnel.

As can be seen from Figure 15, when the HSR is running normally outside the tunnel, the average grayscale of the image only fluctuates in a very small range, and basically remains relatively stable. When the HSR enters the tunnel, the average gray value of the captured image drops to about 5 (as shown in Figure 13b, the image is basically black) because the fill light is not yet turned on and the light inside and outside the tunnel changes drastically. As the fill light is turned on, after a short period of time to adapt to the HSR will remain in a stable state in the tunnel and continue to travel, the average gray scale of the image will remain relatively stable again (as shown in Figure 13d, the image is basically all white) and the time of the HSR in the tunnel is determined by the speed of the HSR and the length of the tunnel. When the HSR out of the tunnel, due to run from a relatively dark environment to a bright environment, the HSC overexposure phenomenon will occur. At this time the average gray scale of the HSC captured by the image will jump to close to 250 or so.

### 4.3. Sun Detection Algorithm Based on Local Average Grayscale of Image Pantograph Region

**Figure 16:**
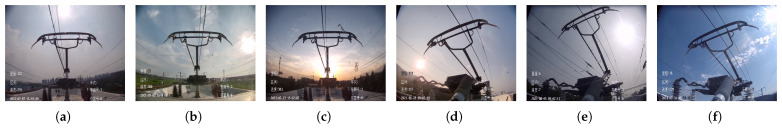
Sun did not affect YOLO detection of pantographs in HSR-A and HSR-B. (**a**) Case I. (**b**) Case II. (**c**) Case III. (**d**) Case IV. (**e**) Case V. (**f**) Case VI.

The influence of the sun on the HSR is full of uncertainty. We cannot accurately predict that a HSR happens to pass by at a certain time on a certain line, and the sun also happens to appear in the pantograph imaging region of the HSR at this time, and affect YOLO’s assessment of the pantograph state. Moreover, not all suns are as jealous of pantograph detection as shown in Figure 11. Figure 16 shows the situation where the sun appears in some images taken by HSC, but the sun does not affect YOLO’s detection of the pantograph region.

**Figure 17:**
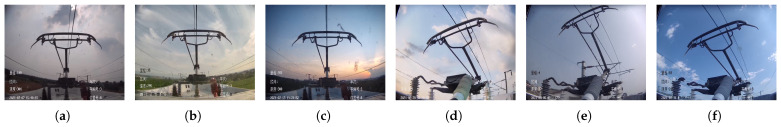
The corresponding HSC in Figure 16 captures the scene without the sun in the frame. (**a**) Case I. (**b**) Case II. (**c**) Case III. (**d**) Case IV. (**e**) Case V. (**f**) Case VI.

**Figure 18:**
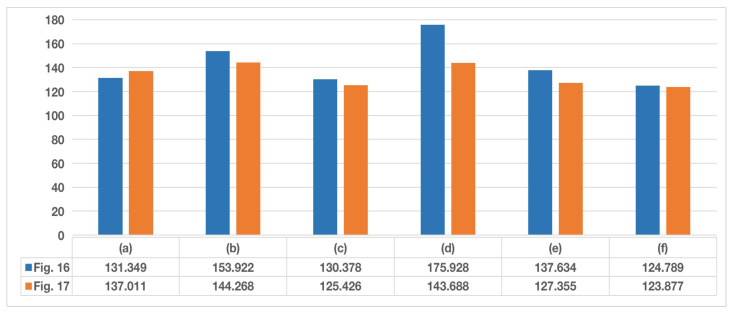
Average grayscale comparison.

The screen of the corresponding scene in Figure 16 after the high speed rail leaves the area affected by the sun is shown in Figure 17. Furthermore, the average grayscale of the corresponding scenes in Figure 16 and Figure 17 is shown in Figure 18.

**Figure 19:**
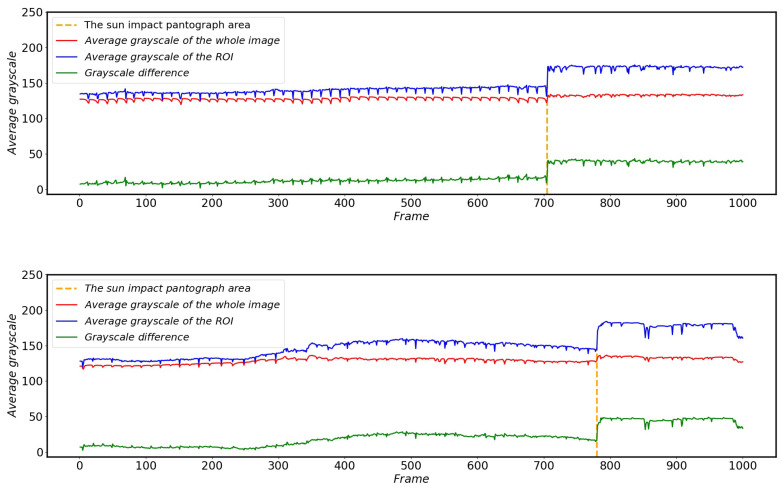
Average grayscale variation in the corresponding areas of HSR-A (**top**) and HSR-B (**bottom**) during sun influence pantograph detection.

It can be found that the overall average grayscale of the image is not necessarily increased after the sun appears in the image captured by the HSC. However, when the sun affects the detection of pantographs, it will definitely cause an increase in the average grayscale of ROI. When the sun is not present the difference between the overall image and the average grayscale of the ROI is not significant, but once the sun affects the pantograph, it will definitely cause a large difference between the two. Using this unique difference, it is possible to determine whether the pantograph is detected as anomalous in the current image due to the sun. When the sun affects the pantograph detection, the average grayscale change of the overall image and ROI and the corresponding difference between the two average gray levels are shown in Figure 19.

### 4.4. Background Detection Algorithm for Catenary Support Devices, Bridges, and Platforms Based on Vertical Projection

**Figure 20:**
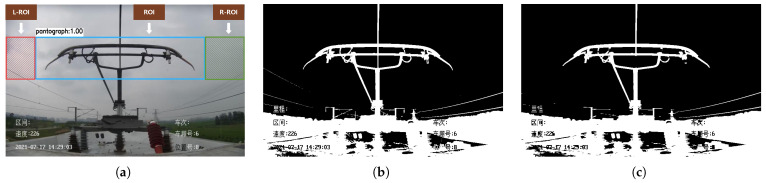
Image binarization and opening operations. (**a**) L-ROI, ROI and R-ROI. (**b**) Binary image. (**c**) Binary image after opening operation.

**Figure 21:**
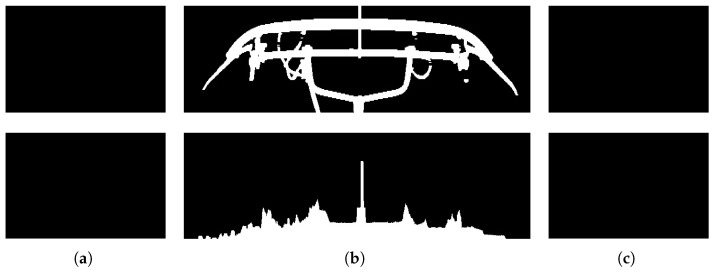
Binary image of different regions and the corresponding vertical projections after the opening operation. (**a**) L-ROI. (**b**) ROI. (**c**) R-ROI.

Catenary support devices, bridges, and platforms do not have an excessive effect on the average grayscale of the images captured by the HSC, so for these three common external disturbances, the choice was made to eliminate the relevant interference by using vertical projection. As shown in Figure 20a, based on the ROI positioned by YOLO V4, the left region of interest (L-ROI) and right region of interest (R-ROI) can be positioned. Firstly, the image captured by the HSC is binarized to highlight the object to be studied, and the result of binarization is shown in Figure 20b. Then the binary image is passed through the image to reduce the interference in the image by the opening operation, and the image after the opening operation is shown in Figure 20c. Finally, the vertical projection of the L-ROI, ROI, and R-ROI regions is calculated by the result of the open operation as shown in Figure 21, where the height of the white region of the vertical projection reflects the number of pixels in the white region on the corresponding horizontal coordinates in the binary image.

**Figure 22:**
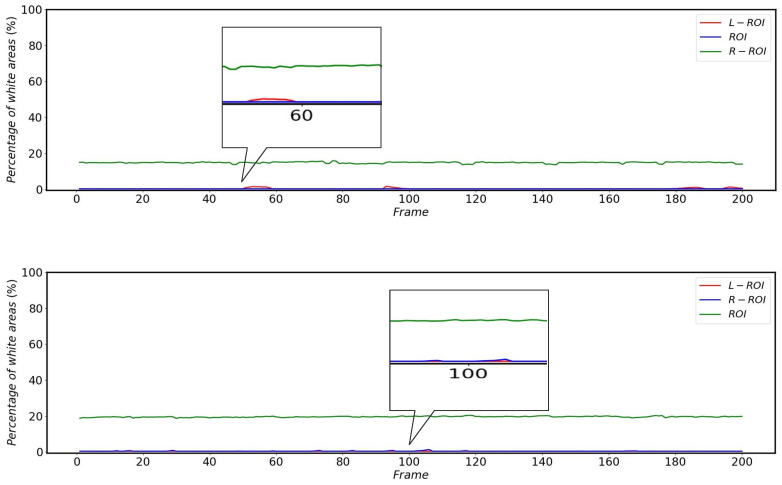
Change in the percentage of white areas in the vertical projection of different areas of HSR-A (**top**) and HSR-B (**bottom**) when the HSR is operated without external disturbances.

As shown in Figure 22, the percentage of white areas in the vertical projections of L-ROI and R-ROI is low when the HSR is operating normally without external disturbance, while there is a large percentage of white areas in the vertical projections corresponding to ROI.

**Figure 23:**
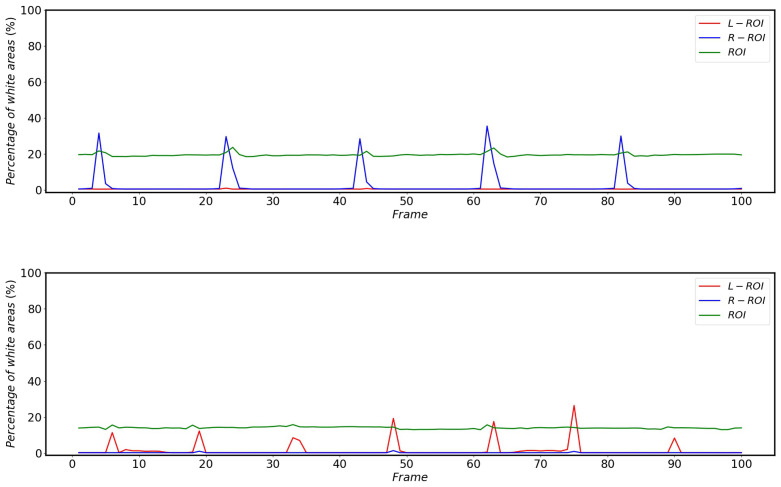
Changes in the percentage of white areas in the vertical projections of different areas of HSR-A (**top**) and HSR-B (**bottom**) during HSR operation after being affected by the catenary support devices.

The impact of the catenary support device on the pantograph detection is much smaller compared to other complex backgrounds, but the percentage of white areas in the vertical projection still reflects the changes brought about by this scenario very accurately. The changes in the percentage of white areas in the vertical projection after different areas in the L-ROI, ROI and R-ROI are affected by the catenary support devices during the operation of the HSR are shown in Figure 23.

**Figure 24:**
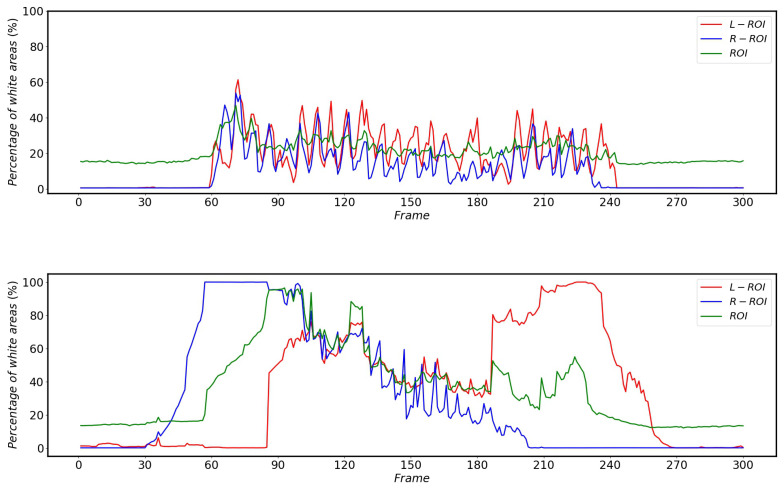
Changes in the percentage of white areas in the vertical projections of different areas of HSR-A (**top**) and HSR-B (**bottom**) during HSR operation after being influenced by the bridge.

The effect of bridges on the percentage of white areas in the vertical projection of different regions during HSR operation is shown in Figure 24. Since the HSC angles of HSR-A and HSR-B are different, the bridges do not have the same effect on the percentage of white in the vertical projection areas of L-ROI and R-ROI, but both cause at least one of the L-ROI or R-ROI to have a huge change in the percentage of white area in the vertical projection.

**Figure 25:**
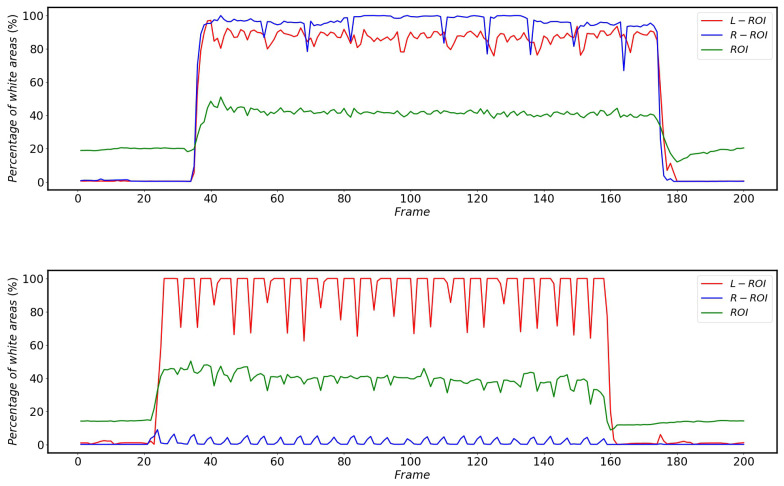
Changes in the percentage of white areas in the vertical projections of different areas of HSR-A (**top**) and HSR-B (**bottom**) during HSR operation after being influenced by the platform.

The effect of the platform on the percentage of white areas in the vertical projection of the different areas is shown in Figure 25. Furthermore, due to the HSC angle, the impact of the platform on HSR-A and HSR-B is different, but both have an impact on at least one of the R-ROI or L-ROI.

From Figure 22, Figure 23, Figure 24 and Figure 25, it can be seen that the percentage of white area in the projection corresponding to ROI does not change much when subjected to complex background interference, while the changes of L-ROI and R-ROI are very obvious after subjected to complex background interference, so this paper mainly detects the presence of complex background interference by the projection of L-ROI and R-ROI areas.

### 4.5. Overall Process of HSR Complex Background Detection Algorithm

**Figure 26:**
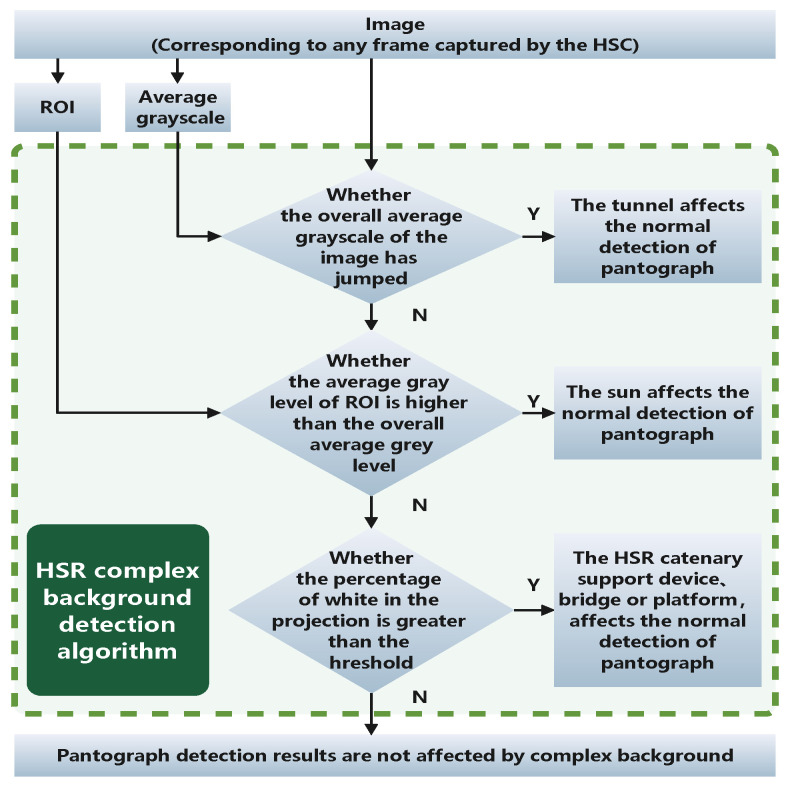
HSR complex background detection algorithm process flow chart.

The overall process of the complex background detection algorithm is shown in Figure 26. For a pantograph image captured by a HSC, when it cannot be detected or is detected as abnormal, the complex background detection algorithm is needed to assess whether the current detection result has the possibility of being affected by the complex background.

The specific process is as follows: First, the change of the average grayscale of the current image as a whole and the average grayscale of the previous frame as a whole is used to evaluate whether the detection result may be affected by the drastic change of light before and after the HSR enters and leaves the tunnel. If not, the relationship between the overall average grayscale of the image and the average grayscale of the ROI is used to assess whether the sun may have intruded into the pantograph region and thus influenced the pantograph detection. If the influence of the sun can still be excluded, the detection of the catenary support devices, platforms, and bridges is achieved by vertical projection to finally determine whether the pantograph detection results are influenced by the complex background at this time.

If the influence of complex background on the detection result is excluded by HSR complex background detection algorithm, then there are still two possibilities for the pantograph not to be detected or detected as abnormal: (1) although the current image is not disturbed by complex background, it may be disturbed by other interference which leads to misjudgment of the pantograph, (2) the pantograph does appear abnormal. In this case, the overall algorithm proposed in Section 5.1 of this study is combined to achieve accurate detection of the real situation of pantographs.

## 5. Experiments and Conclusions

### 5.1. The Overall Process of Pantograph Detection Algorithm

**Figure 27:**
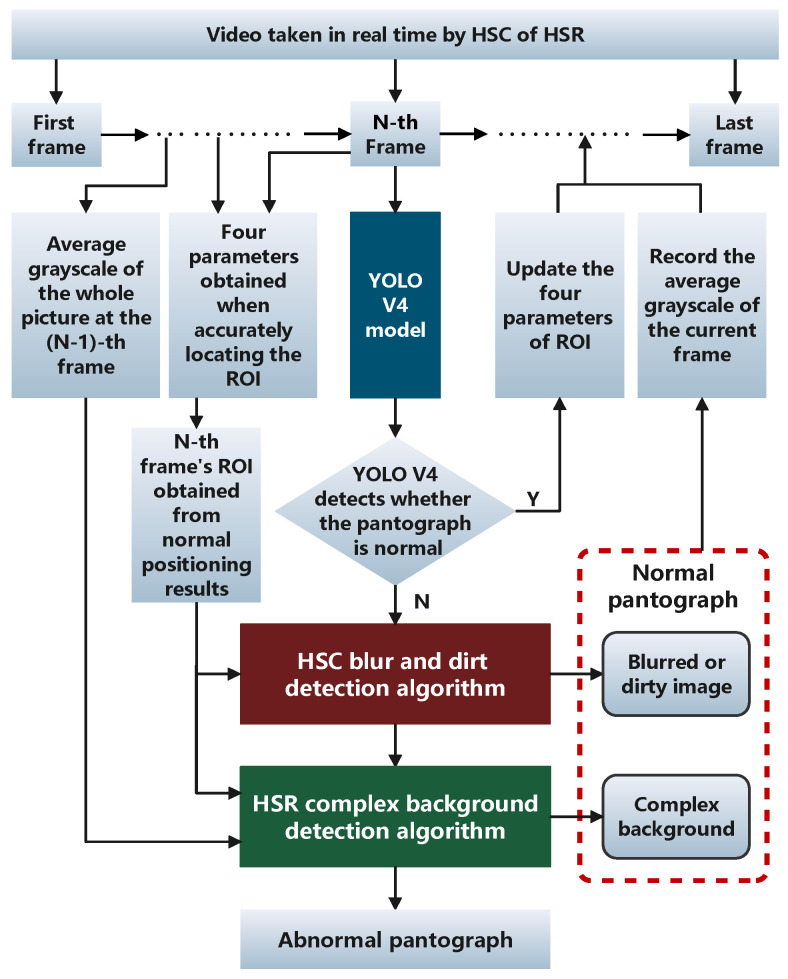
Pantograph detection algorithm process flow chart.

The overall process of the algorithm is shown in the Figure 27, when YOLO V4 cannot detect the pantograph in a frame or detect it as abnormal, the algorithm gives priority to detecting it through the HSC blur and dirt detection algorithm, and when the detection abnormality is ruled out as a result of dirty or blurred screen, then the HSR complex background detection algorithm to determine whether the detection of abnormalities is caused by complex background. Finally, we can realize the accurate judgment of the pantograph state.

### 5.2. Performance Evaluation of Algorithms under Complex Background Interference

The operation of HSR requires frequent face to the interference and influence brought by scenarios such as catenary support devices, sun, bridges, platforms, and tunnels to pantograph detection. The performance of different methods in detecting pantographs in complex backgrounds is shown in Table 1.

Refs. [12,17,18,38,39,40] all proposed good methods and ideas in order to improve the performance of their respective algorithms in complex backgrounds. However, in the face of more complex background disturbances and effects during the actual operation of HSR, the relevant algorithms still cannot achieve correct detection of pantographs under these complex backgrounds. In contrast, the HSR complex background detection algorithm proposed in this study can well achieve the correct detection and evaluation of the pantograph state under the relevant scenes. The results in Table 1 show that the method proposed in this study is more suitable for the real situation and practical needs of HSR, and performs better under the influence of complex background.

### 5.3. EOR-Brenner Evaluates the Sharpness of Pantograph Images Captured by HSC

**Figure 28:**
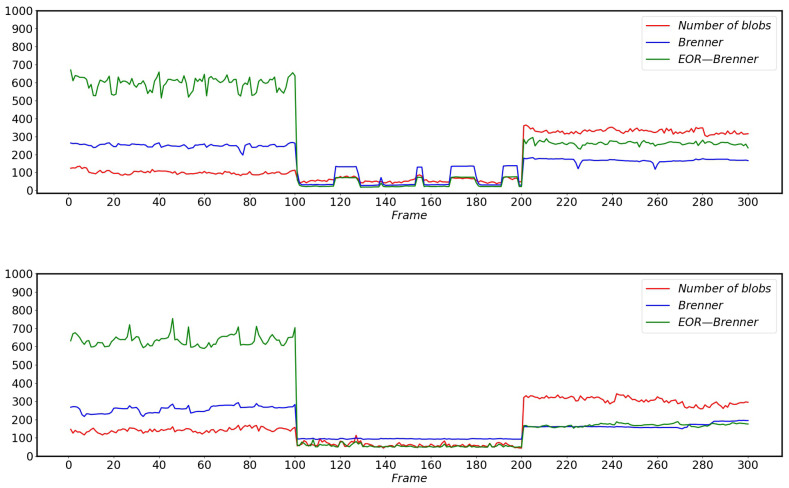
EOR-Brenner evaluation results of images captured by HSR-A and HSR-B under different conditions.

Figure 28 shows the scores of EOR-Brenner on the sharpness of the images captured by two different models under different conditions. Where Frame 1–Frame 100 corresponds to the images captured by HSC during normal operation without any disturbance, Frame 101–Frame 200 corresponds to the blurred image caused by rain affecting the HSC, and Frame 201–Frame 300 is the dirty HSC lens.

Comparing Figure 28, it can be seen that EOR-Brenner gives higher scores than Brenner for clear pantograph images; for blurred pantograph images EOR-Brenner gives lower scores than Brenner for image sharpness; and the scores are very close when dirty. At the same time, EOR-Brenner has higher distinguishability between clear, blurred and dirty images, while the scores of the original Brenner images are very similar when they are dirty and clear. The improved EOR-Brenner algorithm is more in line with the real operating environment of HSR and better meets the actual needs of HSR operation.

### 5.4. Evaluation of the Overall Performance of the Algorithm in This Study

The combined test results for complex scenes and blurred and dirty cases are shown in Table 2 and Table 3. The red part corresponds to a clear image without interference, the gray part corresponds to a blurred image, the purple part corresponds to an image affected by dirt, and the pink part corresponds to an image disturbed by a complex environment.

**Figure 29:**
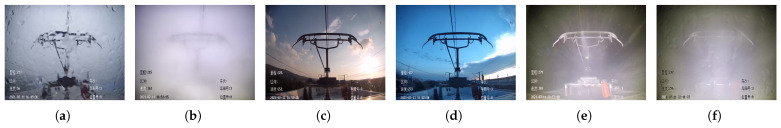
Scenes taken at different moments of the same HSR in rainy weather. (**a**) Case I. (**b**) Case II. (**c**) Case III. (**d**) Case IV. (**e**) Case V. (**f**) Case VI.

Figure 29 shows the scene of the same HSR running at different times on the same line. Due to the intermittent heavy rainfall, the blurring of the images caused by the HSC affected by rain at different moments is not the same. For the same train on the same line when it is affected differently the results of the clarity algorithm for it are shown in Table 4.

As can be seen from Table 2, Table 3 and Table 4, regardless of the cases in which different complex backgrounds or external disturbances affect the pantograph detection of different HSR, or the cases in which the same HSR affects the pantograph detection at different moments due to changes in the external environment, the EOR-Brenner algorithm proposed in this study can accurately evaluate the sharpness of these pantograph images under the influence of disturbances, and the clearer the image, the higher the score. For the blurred pantograph images, the EOR-Brenner algorithm scores them much lower than the normal pantograph images, so as to achieve an accurate judgment of the blurred situation. However, it should be noted that for the images corresponding to Figure 6 when the HSC lens is dirty, a large number of blobs appear on the lens due to the dirt, which will make the image have more edge details at this time, so the EOR-Brenner does not score the dirty image low. However, the number of blobs on the dirty image is much higher than the pantograph images in other cases, so the number of blobs can achieve accurate detection of dirty images.

For the case of complex background affecting pantograph detection, comparing Table 2 and Table 3, we can see that the average gray scale of the whole image (Figure 13) before and after entering and leaving the tunnel will suddenly jump to around 0 or 255, while other disturbances affecting the pantograph will not lead to such a drastic change in gray scale value, through this jump in gray scale value can provide a strong basis for whether the high speed rail is driving into the tunnel, so as to exclude the high speed rail The effect on pantograph detection when entering and leaving the tunnel. When the sun affects the pantograph detection (Figure 11) it causes a large difference between the average grayscale of the ROI and the average grayscale of the whole image, while in other cases the difference between the average grayscale of the pantograph area and the whole image is small. Compared with other disturbances, contact network support devices, bridges, and tunnels, when affecting pantograph detection (Figure 10, Figure 12 and Figure 14), cause the white percentage of the vertical projection of at least one of the L-ROI region and R-ROI region to reach more than 35%, while the percentage of the vertical projection of the L-ROI and R-ROI regions in other scenes basically remains around 1%, with the maximum not exceeding 10%. Accurate detection of these scenes can be achieved by this feature.

The results of the comprehensive test for a variety of scenes at the same time are shown in Table 5. Meanwhile, we demonstrate the effectiveness of each module by the ablation experiments shown in Table 6. It is easy to find that the HSR complex background detection algorithm and HSC blur and dirt detection algorithm proposed in this study can greatly improve the accuracy of pantograph inspection evaluation when complex background and external disturbance exist. In general, the algorithm proposed in this study is in line with the real situation of HSR operation and meets the actual needs of HSR operation, which has a greater practical application value.

## 6. Conclusions

The pantograph detection algorithm proposed in this study fully considers the actual needs of HSR operation, and at the same time conducts a comprehensive and synthesize analysis of the complex scenarios and external disturbances that need to be faced during HSR operation. The proposed algorithm achieves precision of 99.92%, 99.90% and 99.98% on different test samples. At the same time, for three different samples, the processing speed of the algorithm per second reaches 49 FPS, 43 FPS and 43 FPS respectively, which meets the requirement of the algorithm to process at least 25 images per second in the actual operation of HSR. This method solves two major difficulties when using neural network to realize pantograph detection: firstly, the current pantograph detection method is easily affected by external interference, and cannot detect and eliminate external interference. Secondly, because the pantograph samples in complex situations are few and difficult to collect, the sample set for training the neural network cannot cover all situations, so the detection accuracy in complex situations is low.

## Figures and Tables

**Table 1:** Performance of different algorithms when dealing with complex backgrounds.

Method	TM	MS + SIFT	MS + KF	PDDNet	SED	Improved Faster R-CNN	The Method of This Study
	[38]	[39]	[40]	[12]	[17]	[18]	
Whether the pantograph can be detected correctly under the complex background	×	×	×	×	×	×	✓

**Table 2:** Comprehensive evaluation of the images presented in this article I.

Image Serial Number	Different Sharpness Evaluation Algorithms								
	Tenengard [41]	Laplacian [42]	SMD [43]	SMD2 [44]	EG [45]	EAV [46]	NRSS [47]	Brenner [33]	EOR -Brenner
Figure 2 left	22.5	4.24	1.81	2.01	9.34	38.18	0.79	252	704
Figure 2 right	31.1	8.25	3.23	5.18	17.26	48.25	0.91	400	876
Figure 5a	9.4	2.18	0.76	0.57	2.31	23.44	0.75	95	55
Figure 5b	10.57	2.49	0.86	0.64	2.46	27.89	0.75	117	64
Figure 6a	31.64	4.45	2.72	2.35	13.92	39.01	0.82	158	228
Figure 6b	32.81	5.52	2.77	2.75	16.32	50.55	0.84	286	476
Figure 10a	26.27	4.55	2.13	2.48	11.98	44.48	0.77	269	686
Figure 10b	39.79	6.76	3.54	5.13	21.42	66.29	0.81	363	767
Figure 11a	24.00	4.56	2.20	2.71	13.62	51.25	0.81	143	310
Figure 11b	14.00	2.54	1.22	1.42	6.77	42.21	0.78	75	285
Figure 12a	42.92	6.78	3.47	3.96	21.19	56.17	0.79	358	613
Figure 12b	31.82	4.84	2.67	3.61	17.03	55.23	0.78	221	346
Figure 13a	27.18	4.12	2.30	2.75	13.49	46.28	0.76	162	356
Figure 13b	10.44	2.21	0.86	0.85	2.43	9.76	0.74	229	230
Figure 13c	20.96	3.70	1.80	1.54	7.97	32.38	0.75	209	342
Figure 13d	10.65	2.34	0.88	0.74	2.38	10.11	0.75	245	246
Figure 14a	46.62	7.53	4.05	6.12	26.28	80.26	0.78	305	924
Figure 14b	39.25	6.14	3.38	3.21	22.02	86.59	0.78	310	551

**Table 3:** Comprehensive evaluation of the images presented in this article II.

Image Serial Number	Vertical Projection		Average Grayscale		Number of Blob
	L-ROI (%)	R-ROI (%)	Whole	ROI	
Figure 2 left	0.5	0.5	135	146	57
Figure 2 right	0.3	0.4	148	154	62
Figure 5a	0.4	0.4	159	175	30
Figure 5b	0.5	0.3	158	179	29
Figure 6a	3.3	1.1	179	190	481
Figure 6b	6.1	0.7	143	149	445
Figure 10a	1.9	38.6	120	114	61
Figure 10b	14.1	72.0	117	116	73
Figure 11a	3.4	0.5	178	212	69
Figure 11b	0.2	0.5	189	221	44
Figure 12a	46.0	44.7	118	122	140
Figure 12b	83.2	67.7	106	100	91
Figure 13a	47.8	69.0	149	154	117
Figure 13b	0	0	2	0	26
Figure 13c	0.5	0.5	52	55	61
Figure 13d	0.5	0.5	250	252	45
Figure 14a	94.3	99.6	112	118	130
Figure 14b	100	7.9	127	141	106

**Table 4:** Performance of the same HSR at different times with different levels of disturbance.

Image Serial Number	The Actual Time Corresponding to the Scene	Different Sharpness Evaluation Algorithms								
		Tenengard [41]	Laplacian [42]	SMD [43]	SMD2 [44]	EG [45]	EAV [46]	NRSS [47]	Brenner [33]	EOR-Brenner
Figure 29a	16:49:36	16.30	3.15	1.31	1.09	5.69	32.18	0.77	124	149
Figure 29b	16:51:45	9.16	2.45	0.74	0.54	2.20	28.28	0.74	125	63
Figure 29c	18:59:35	22.53	4.72	1.79	1.73	7.98	46.70	0.78	256	756
Figure 29d	19:22:54	23.29	4.82	1.90	1.93	9.12	40.97	0.79	235	764
Figure 29e	20:57:08	9.46	1.76	0.82	0.69	3.45	29.17	0.76	50	81
Figure 29f	22:41:23	9.94	2.37	0.85	0.62	2.54	32.92	0.74	112	59

**Table 5:** Overall algorithm testing.

Serial Number	Type of Sample	Number of Samples	Total Algorithm Run Time	FPS	Precision
I	Complex backgrounds only	14,985	304 s	49	99.92%
II	Complex backgrounds + Blur	14,999	346 s	43	99.90%
III	Complex backgrounds + Dirt	14,974	349 s	43	99.98%

**Table 6:** Impact of different modules on the overall algorithm.

	Precision-I	Precision-II	Precision-III
The complete algorithm proposed in this study	99.92%	99.90%	99.98%
− HSR complex background detection algorithm	73.97%	84.76%	85.32%
− HSC blur and dirt detection algorithm	96.24%	73.16%	77.13%
− HSR complex background detection algorithm and HSC blur and dirt detection algorithm	70.36%	57.42%	63.10%

## Data Availability

Not applicable.
